# The vaginal microbial signatures of preterm birth woman

**DOI:** 10.1186/s12884-024-06573-1

**Published:** 2024-06-14

**Authors:** Huan Li, Mengzhen Han, Junnan Xu, Na Li, Hong Cui

**Affiliations:** 1 Department of Obstetrics and Gynecology, Research Center, Shengjing Hospital of China Medical University, China Medical University Birth Cohort, Shenyang, 110004 China; 2 Department of Breast Medicine, Cancer Hospital of China Medical University, Liaoning Cancer Hospital, Shenyang, China

**Keywords:** Preterm birth, Vaginal microbiome, 16S rRNA-seq, ROC, WGS

## Abstract

To explore the differences of vaginal microbes in women with preterm birth (PTB), and to construct prediction model. We searched for articles related to vaginal microbiology in preterm women and obtained four 16S rRNA-sequence datasets. We analyzed that for species diversity and differences, and constructed a random forest model with 20 differential genera. We introduce an independent whole genome-sequencing (WGS) data for validation. In addition, we collected vaginal and cervical swabs from 33 pregnant women who delivered spontaneously full-term and preterm infants, performed WGS in our lab to further validate the model. Compared to term birth (TB) samples, PTB women vagina were characterized by a decrease in *Firmicutes*, *Lactobacillus*, and an increase in diversity accompanied by the colonization of pathogenic bacteria such as *Gardnerella*, *Atopobium* and *Prevotella*. Twenty genus markers, including *Lactobacillus*, *Prevotella*, *Streptococcus*, and *Gardnerella* performed well in predicting PTB, with study-to-study transfer validation and LODO validation, different gestation validation showing good results, and in two independent cohorts (external WGS cohorts and woman samples WGS cohorts) in which the accuracy was maintained. PTB women have unique vaginal microbiota characteristics. A predictive model of PTB was constructed and its value validated from multiple perspectives.

## Importance

This study integrates current data on the vaginal microbiome of women with preterm birth to comprehensively investigate the unique vaginal microbiome of women with preterm birth. A preterm birth risk prediction model based on 20 characteristic genera was constructed, and its effectiveness was verified by internal, external, and collected whole genome sequencing data.

## Introduction

Preterm birth (PTB) is commonly defined as delivery at less than 37 weeks of gestation and is a significant cause of neonatal death worldwide [1], accounting for 75% of perinatal mortality. Risk factors for PTB include infection [2–4], advanced maternal age, history of PTB, and maternal stress [5], and may be the result of a single or multiple risk factors combined for adverse outcomes. Although the health status of preterm infants and pregnant women has improved significantly with the availability of medical technology, the disability and even death is still not negligible. The health problems are not limited to the birth and infancy stages, but may continue throughout the preterm child development and all life [6, 7]. In addition, PTB also affects the physical and psychological health of pregnant women [8], and a poor pregnancy outcome can have a detrimental effect on the psychological stress of subsequent births, thus demonstrating the importance of PTB control.

As disease research has moved from the macroscopic to the microscopic, the existence of a correlation between dysbiosis of the human microbial environment and disease occurrence has been widely recognized. Vaginal microbes (VM) as a female-specific microbial community are closely linked to the stability and health of reproductive tract. The predominance of *Lactobacillus* often symbolizes a healthy VM environment [9, 10], while the decrease is associated with dysbiosis and infections [11]. It has been revealed that elevated estrogen during pregnancy stimulates the accumulation of glycogen in the vaginal epithelium, which acts as a source of carbohydrates to facilitate the colonization of *lactobacilli* and provides a protective effect [12]. In contrast, deficiency of *Lactobacillus* is associated with increased odds of short cervix [13], and short cervical length is one of the strongest predictors of spontaneous PTB [14]. The association between reproductive tract infections and the risk of PTB has been extensively studied in recent years. About 25% of PTB are attributed to intrauterine infection and subsequent immune response [5]. It has been demonstrated that there is a degree of sharing of microbes between the vagina and the uterus [15, 16]. The presence of microbes isolated from amniotic fluid or amniotic membranes in PTB women in the lower genital tract [17, 18] suggests that specific VM may traveling up the genital tract to the uterus as an infectious agent. In addition, bacterial vaginosis (BV) is a risk factor of PTB [19, 20], which increases the risk twofold. However, it is not possible to determine that healthy women without infection are not at risk. It is known that the VM of pregnant women differ from those of non-pregnant women [21], and that the process of pregnancy itself alters the microbial environment, due to endocrine influences, suggesting that normal women with specific microbes or specific microbial environments are also at risk of PTB.

Currently, there is no comprehensive system for predicting PTB in clinical practice and individual differences are ignored. Reducing the incidence of PTB requires prediction and interventions at earlier gestation and even in the preparatory phase. The development of non-invasive, low-cost, and controllable microbiological tools is necessary. In addition, the correlation studies between VM and PTB do not have good consistency [22, 23].

In this study, we integrated VM 16S rRNA-seq from different regions, involving 337 samples from 4 studies, including 181 PTB women and 156 TB women. A comprehensive and multidimensional analysis of VM in PTB women was performed. A high-precision PTB prediction model was also constructed and its applicability was tested in different gestational periods. We also collected 33 whole genome sequencing (WGS) and incorporated a WGS data to validate the model. In addition, co-abundance analysis and Kyoto Encyclopedia of Genes and Genomes (KEGG) functional prediction analysis were performed. In conclusion, the aim of this study was to investigate the possible association between VM and PTB and to understand the potential mechanism, with the aim of providing some theoretical basis for the future development of noninvasive prediction and intervention.

## Method

### Study participants and sampling

We performed a prospective cohort study of recruiting 33 women with and without risk factors for PTB between August 2020 and December 2020. The study was approved by Ethics Service Committees of Shengjing Hospital of China Medical University (EC number:2017PS318K). All ethical guidelines for human research were followed and participants provided written informed consent. Inclusion criteria were women over 18 years of age and pregnant. Exclusion criteria included women under 18 years of age, multiple pregnancy, and sexual intercourse or antibiotic treatment within 72 h of sampling and HIV or Hepatitis C positive status. In our own sampling, women were recruited upon presentation to the third trimester unit during birth surveillance clinic (28–37^+ 6^ weeks gestation). Following informed consent, a high vaginal swab was taken using a speculum from the posterior vaginal fornix as VM sample. The samples delivered to the laboratory within 4 h.

### DNA extraction and purification

One millilitre of sterile phosphate-buffered saline (pH = 7.4) was added to each swab followed by rigorous vortexing for 30 s. Total DNA was extracted using the QIAamp DNA Mini kit (QIAGEN, 51,304) and manufacturer’s instructions. Briefly, 1000 µl of swab material was centrifuged to collect the precipitate, which was suspended with 500 µl of phosphate-buffered saline, followed by mechanical (Tissuelyser-24, Shanghai Jingxin) physical grinding to break up the cells, and then treated with chemical lysis solution AL 200 µl and 20 µl proteinase K to disrupt the pellet. DNA was eluted with 50 µl elution buffer and DNA concentration was determined using the Qubit High Sensitivity Kit according to the manufacturer’s instructions, and samples were stored at -20 °C. Finally, all samples were normalized to 100 ng. Shotgun metagenomic sequencing Libraries of DNA were prepared according to standard Vazyme protocols. Briefly, DNA was sheared by heating to 37 °C for 15 min. Sequences tags were added and amplification occurred for 3–4 cycles before samples were purified with AMPure magnetic beads. DNA was quantified by Qubit dsDNA HS assay kit. The short double-stranded DNA was then denatured and cyclized, and finally made into nanospheres for on-board sequencing. A sample of sterile water was processed in parallel with the DNA during library preparation to act as a negative control. Libraries were sequenced using 250 bp paired-end kit on the MGI 2000RS platform.

### Public data collection

We collected data from published studies in PubMed.gov containing public available 16S rRNA-seq data on patients with PTB and TB. Raw sequencing data of these studies were downloaded using Ascp (v) from Sequence Read Archive (SRA) and European Nucleotide Archive (ENA) using identifiers: PRJEB43005 [24]、PRJNA725416 [25]、PRJDB10581 [26] and PRJNA687274 [27]. In addition, one additional cohorts from shotgun metagenomic sequencing (PRJEB34536) [28] was also added as independent cohorts for confirmatory analysis.

### Data preprocessing

Clean reads were obtained from the raw sequencing data using VSEARCH (v2.18.0) [29], as follows. The paired-end reads were merged using default parameters. All sequences were trimmed by using VSEARCH according to different sequencing region. Sequences with zero mismatches were extracted and an error rate for the overlap of > 0.1 were discarded. After dereplicating and denoising, according to the VSEARCH operational taxonomic unit (OTU) analysis pipeline, identifying representative sequences form unique sequences. Then, OTUs were clustered based on 97% sequence identity. Taxonomy classification was assigned based on the naive Bayes classifier using the VSEARCH against the rdp_16s_v16 reference sequences [30]. After removing Chloroplast from taxonomy, the classification from phylum to genus level was further identified on a Bayesian Lowest Common Ancestor (LCA) method [24].

### Community state type analysis

The community state types (CST) was proposed by Ravel et al. [31] and later supplemented by Gajer et al. [32]. For CST analysis, based on the relative abundance at species level and genus level, using hierarchical clustering with the Jensen-Shannon divergence and Ward linkage to assign each sample [25]. Then, significant differences were analyzed for different CST types in TB and PTB groups using Kruskal–Wallis test. At the same time, the differences of CST classification were compared in alpha diversity index Chao1, Simpson and Shannon.

### Analysis of microbial composition and diversity

The representative sequences obtained from the OTUs were used to calculate sparse distance matrix, then construct evolutionary tree using USEARCH (v11) [33]. Alpha (Shannon-Wiener index, Simpson index and Chao1 index) and beta diversity (Bray–Curtis distance) were calculated at minimum sequence depth with the feature units table. Among them, alpha diversity is estimated using the vegan package (v2.5-7) running in R software v4.0.2, while beta diversity was performed using USEARCH (v11). Subsequently, we performed principal-coordinate analysis (PCoA) based on our Bray-Curtis dissimilarity matrix using the amplicon package (https://github.com/microbiota/amplicon). Finally, the significant differences of PCo1 and PCo2 between different groups were tested using wilcox test and Kruskal–Wallis test [34].

### Difference analysis between OTUs and taxonomy

The significance of differential abundance between TB and PTB groups was tested on a single OTU using a two-sided blocked Wilcoxon rank-sum test implemented in the R (V4.0.2) “amplicon” package (https://github.com/microbiota/amplicon). Differential taxon abundance between TB and PTB groups was performed on normalized abundance data at each taxonomic rank using linear discriminant analysis (LDA) effect size (LEfSe) [35]. Statistical parameters were used with an alpha value of 0.05 for the Kruskal-Wallis/Wilcoxon tests and a threshold of 2.0.

### Co-occurrence and clustering analysis

Correlation relationships between core microbes associated with PTB were determined by co-abundance network analysis [36]. Taxa are represented by different node colors, node degrees are represented by node sizes, and correlations are represented by the width of the connecting lines. Networks were generated by calculating associations between taxa through Spearman correlations (*P* < 0.05, correlation coefficient ≥ 0.7, node degrees > 2). The network was visualized using Gephi (v0.9).

### Model construction and features extraction

To distinguish TB from PTB, we built random forest (RF) models based on OTUs. All the RF models were built using the randomForest R package. And the stratified 10-fold cross-validation was used to configure training and testing data sets. The top features from the top-performing model were selected as “important features” and the top microbial features as “biomarkers” [34]. Finally, all the resulting probabilities served as the input for the pROC R packages to compute the AUC values and draw the receiver operating characteristic (ROC). In order to validate the performance of the important features to differentiate TB from PTB, according to the above analysis method with reference to the published methods [34], we performed study-to-study transfer validation and LODO validation on the entire sample.

To validate the applicability of the model, 2 WGS cohort data were used as independent validation prediction datasets. Data processing was performed using fastp (v0.21.0) to obtain high quality data. Then MEGAHIT (v1.2.9) was used to splice the sequences, and MetaGeneMark (v3.8) was used to perform gene prediction on the spliced sequences, and the redundancy was removed to obtain the unique gene set. Finally, DIAMOND (v0.9.32.133) was used to match to Non-Redundant Protein Sequence Database for species identification. Species abundance was calculated using salmon (v1.4.0), and the applicability of the random forest model was verified based on species abundance.

### Functional profile analysis

The PICRUSt2 software package (https://github.com/picrust/picrust2) can directly predict metagenomic functions based on an arbitrary OTU/ASV table. KEGG were used to detect intergroup enrichment pathways.

## Result

### Samples and characteristics of the data sets

In this study, we firstly investigated public available 16S rRNA-seq data from four studies. In total, we collected 337 samples from pregnancy women (including first trimester: 8–13^+ 6^ weeks gestation; second trimester: 14–27^+ 6^ weeks gestation; third trimester: 28–42 weeks gestation), 181 from TB subjects, and 156 samples from PTB. Total 33 samples with WGS data from Shengjing Hospital of China Medical University and another study with 36 samples of WGS data from public data were identified for verifying the predictive model for PTB.

### Identification of the potential confounder in meta-analysis

**Fig. 1:**
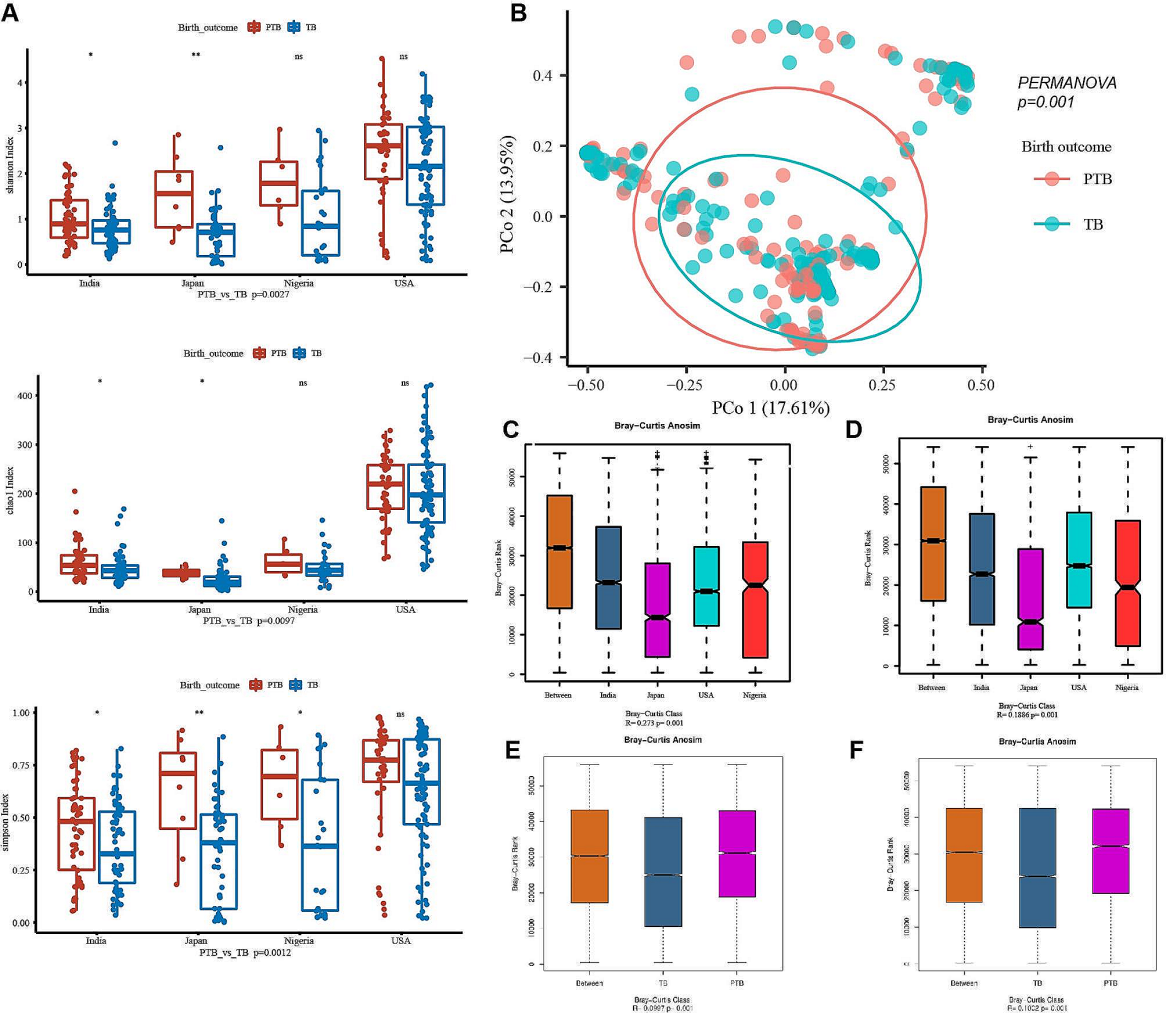
Measures of alpha-diversity and beta-diversity among pre-term birth and full term birth populations. (**A**) Box graphs of mesures of alpha-diversity (chao1 index, simpson index and shannon index) indices of microbial OTUs in each project. (**B**) PCoA based on Bray-Curtis distances for all samples from pre-term birth and full term birth. Elipses represented 95% confidence level. The pink and green ellipses overlapped, indicating insignificant differences between pre-term birth and full term birth cohorts. (**C**) R and p values for beta diversity based on Weighted-Unifrac distances calculated using the Anosim analysis (analysis of Similarities). The closer the R value was to 1, the greater differences between groups were than the differences within groups; the smaller the R value, the less significant the differences between the groups. *p* < 0.05 showed high reliability of the test. The box above “All between Groups” indicated the Weighted-Unifrac distance data of the samples among all groups, while the box above “All within Groups” indicated the Weighted-Unifrac distance data of the samples within all groups. The box below represented the Weighted-Unifrac distance data at phylum and genus levels for different project groups. (**D**) The box below represented Weighted-Unifrac distance data at phylum and genus level for preterm and full-term brith samples. (**E**) Anosim results showed different microbial composition between TB and PTB in phylum level. (**F**) Anosim results showed different microbial composition between TB and PTB in genus level. PTB, Preterm Birth; TB, Term Birth

Due to the differences existed among these studies in both the technical differences and biological differences in four studies, the heterogeneity and confounders of the potential studies was investigated. From the remaining 337 samples, a total of 20,097,432 reads were grouped into 1835 amplicon sequence variants (ASVs). Microbial species contained were significant difference in individuals and ASVs were identified to enlighten the variances by birth outcome. The Chao1 index of alpha diversity was significantly higher in PTB group only in study of “Japan” and “India”, no significantly difference were found in other two studies (Fig. 1A). The Chao1 Index was highest in the study of “USA” among the four studies, however, the it was a little but non-significant difference between PTB and TB cohorts. Moreover, in PTB cohort, the Simpson’s index and the Shannon index, were higher than those in TB cohort, but there was no significant difference in the “USA” cohort (Fig. 1A). In addition, beta diversity indicated PTB and TB almost overlapped and showed insignificant distances for all samples from PTB and TB cohort (Fig. 1B). Weighted-Unifrac distances calculated by the Anosim analysis represented the analysis of similarities. The greater differences among “studies” in phylum level (*R* = 0.273, *P* = 0.001) (Fig. 1C) and in genus level (*R* = 0.1886, *P* = 0.001) (Fig. 1D). Based on Bray-Curtis Anosim analysis, the results suggested there were significant differences between PTB and TB cohorts in phylum level (*R* = 0.097, *P* = 0.001) (Fig. 1E) and in genus level (*R* = 0.1002, *P* = 0.001) (Fig. 1F). R value was more than zero means there were significant difference and the differences were greater among studies than those within PTB and TB groups in phylum level. The factor “study” was demonstrated as a predominant effect on microbial diversity in phylum level.

### CST of term and PTB

**Fig. 2:**
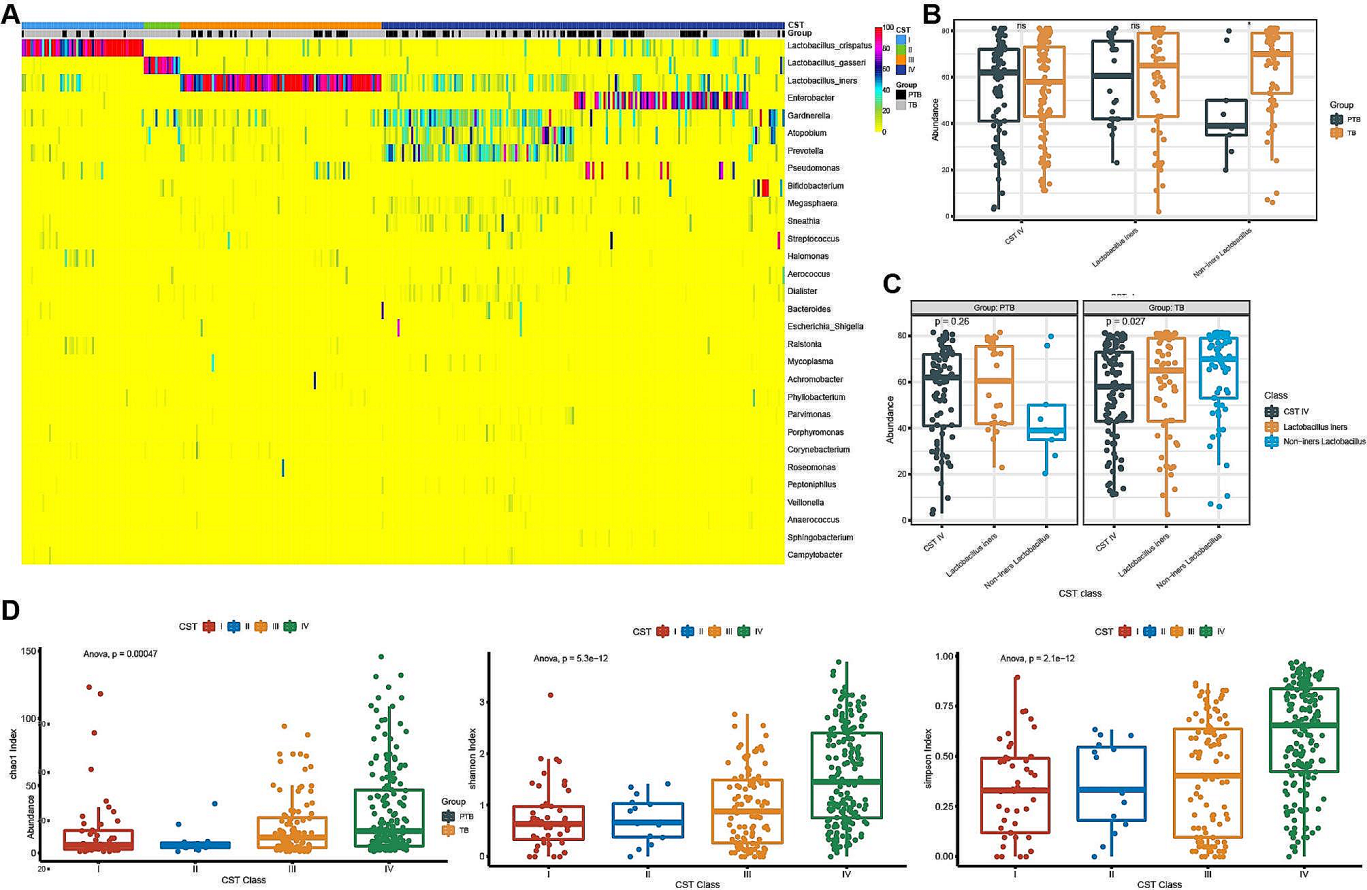
Top 30 taxa and alpha diversity grouped according to CST and birth outcome. (**A**) Heat map of changes in the relative abundance of Top 30 taxa grouped according to CST and birth outcome. (**B**) CST microbiota abundance between PTB cohort and TB cohort. (**C**) Difference microbiota abundance of CST class in PTB group and TB group. (**D**) Box plot Chao1, Simpson, Shannon diversity according to CST category. CST, Community State Type

More than half of the 337 samples were CST IV. Nearly 26.4% had CST III, while just 20.7% classified as CST I and II (Fig. 2A). Because of the small proportion of women with CST I and II, we combined these into a single CST category for statistical analyses (referred to as *non-iners Lactobacillus* CST). According to birth outcome, there were no significant difference in CST IV abundance, *Lactobacillus Iners* abundance and *non*-*iners Lactobacillus* abundance (Fig. 2B). Among the individuals in TB, *Lactobacillus Iners* abundance and *non-iners Lactobacillus* abundance were significantly higher than in CST IV (*P* = 0.041). No significant difference of CST distribution was indicated in PTB group (*P* = 0.67) (Fig. 2C). During the CST categories (CST I, II, III, and IV), box plots of the alpha-diversity indicate that CST IV had significantly higher Chao1 diversity, Simpson index and Shannon index compared to the non-*iners Lactobacillus* CST (Fig. 2D). The results show that CST does not enough classification for birth outcome, and we should focus on the compositional and functional alterations to total VM impact on PTB.

### Alterations of VM composition in PTB

**Fig. 3:**
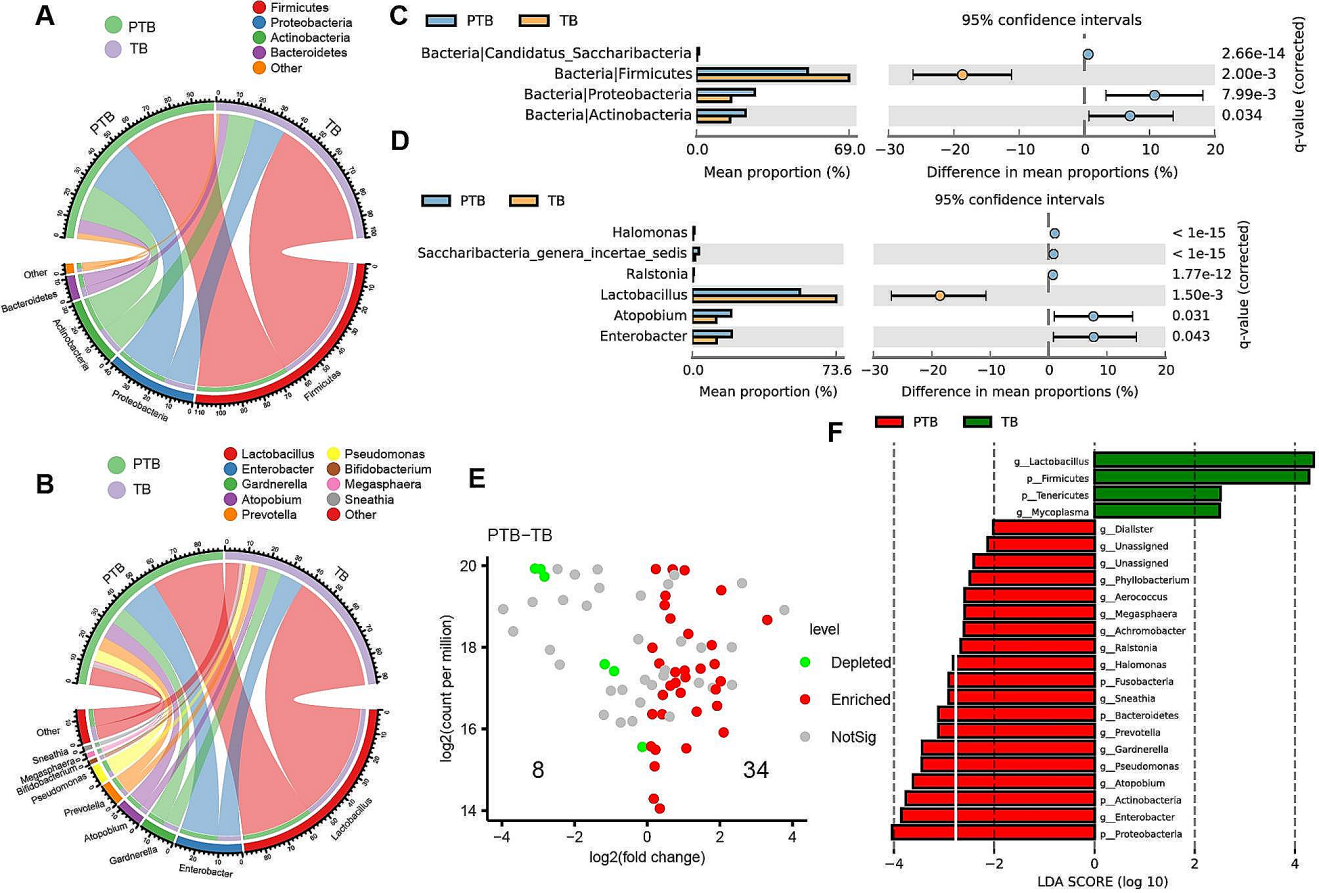
The structure ananlysis of the microbial community between pre-term birth group and full term birt group. (**A**) Cycle graphs of microbial abundance at the phylum level in 337 pregenancy women. (**B**) Cycle graphs of microbial abundance at the genus level in 337 pregenancy women. (**C**) Bar graphs of microbial abuenance of each study at phylum level according to pre-term birth and full term birth category. (**D**) Bar graphs of microbial abuenance of each study at genus level according to pre-term birth and full term birth category. (**E**) Variance explained by birth outcome (pre-term birth versus full term birth) is plotted against for individual ASVs. (**F**) LDA bar graph. Green and red bars represented LDA values for taxa enriched in the pre-term group and those enriched in the full term birth

At the microbiota community phylum and genus level, VM highly varied between PTB and TB cohorts. At the phylum level, the VM was dominated by members of *Firmicutes* and *Proteobacteria*, followed by *Actinobacteria* and *Bacteroidetes* (Fig. 3A). Moreover, the dominant phylum *Fimicutes* and *Preteobacteria* and *Actinobacteria* had significantly decreased abundance (*P* < 0.05) in PTB. At the genus level, results revealed *Lactobacillus*, *Enterobacter*, *Gardnerella*, *Atopobium*, etc. were the dominant genus (Fig. 3B). STAMP were used to identify the difference and analysis the difference in mean proportions between both cohorts with P values. Similarly, *Fimicutes* in phylum level and *Lactobacillus* in genus level were significantly enriched in TB cohort (Fig. 3C). At the genus level, *Proterobacteria*, *Actinobacteria*, *Candidauts*_*Sacchribacteria* and *Atopobium* genus, *Enterobacter* genus enriched in PTB (Fig. 3D). Furthermore, the Wilcox test revealed 42 ASVs with significantly different abundance in the PTB (Fig. 3E). To further investigate the variation of VM in PTB, we performed LEfSe analysis based on the species annotation results. *Proteobacteria*, *Enterobacter*, *Actinobacteria* and 19 other genera were enriched in PTB (Fig. 3F).

### Microbial classification models for PTB

**Fig. 4:**
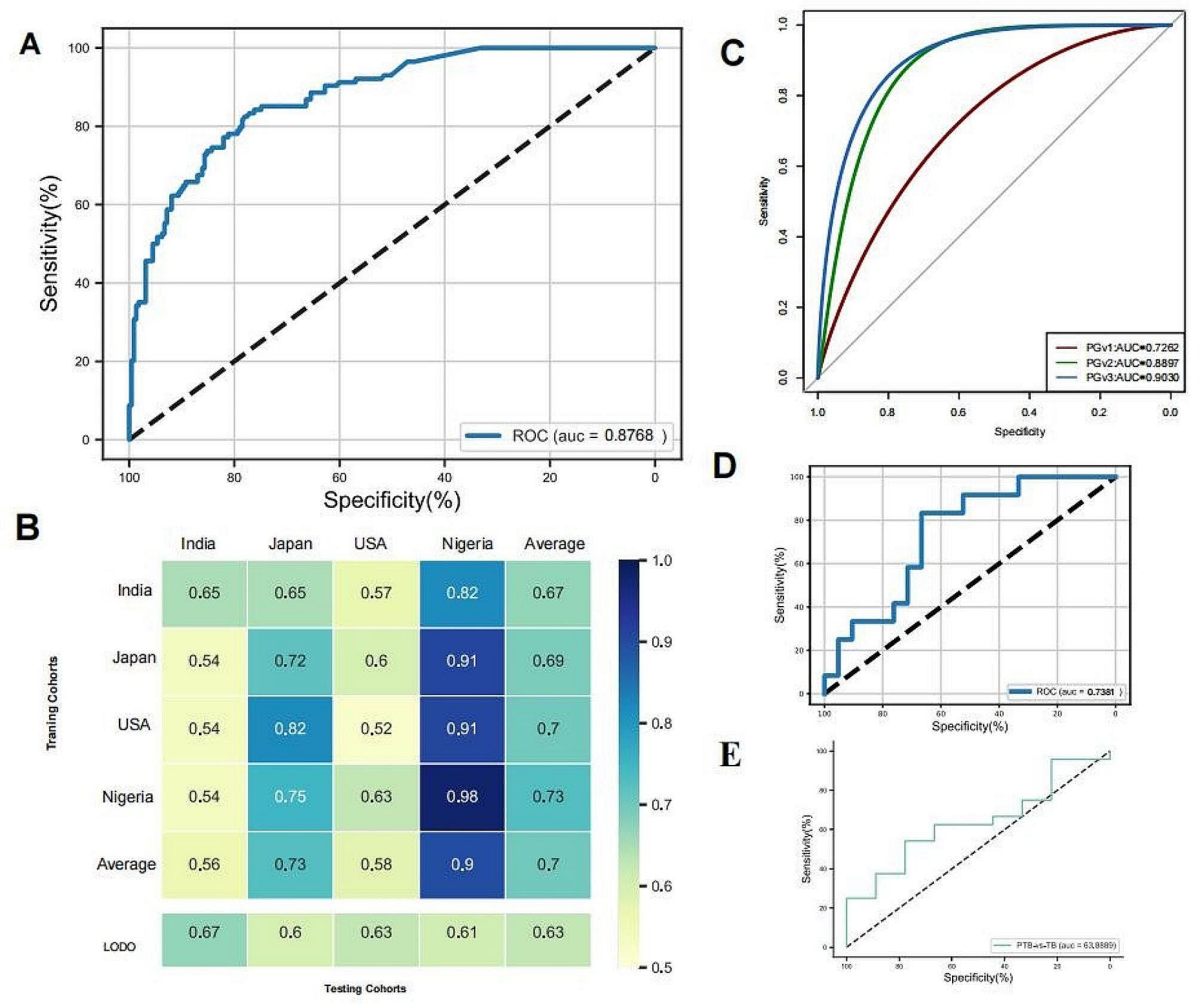
Performance of discriminating pre-term birth from full term birth. (**A**) The AUC of the optimized models constructed with biomarkers in 337 pregenancy women. (**B**) Heat map showing AUROC values for models constructed using genus characteristics in each cohort of the vaginal preterm brith prediction model. (**C**) The AUC of the optimized models constructed with biomarkers in Early visit (8–14 weeks gestation, PGv1), Medium visit (15–24 weeks gestation, PGv2) and Late visit (25–42 weeks gestation, PGv3). (**D**) The validatetion AUC of the 16S rRNA biomarkers model in whole shotgun metagenomics sequencing public cohort. (**E**) Validation AUC value of 16S rRNA biomarker model in whole-genome sequencing of vaginal samples from preterm women

A robust RF model was constructed with a core set of important features, including 20 core differential microbial genera such as *Lactobacillus*, *Prevotella*, *Streptococcus*, *Gardnerella* and *Atopobium* as biomarkers. The model achieved an AUC of 0.88 for distinguishing PTB from TB (Fig. 4A). To test the generalizability and robustness of the identified significant features, we conducted study-to-study transfer validation and LODO validation for all vaginal samples. In the TB versus PTB model, the AUC for the study-to-study transfer validation ranged from 0.52 to 0.98 with a mean of 0.697 (Fig. 4B). The AUC for the LODO analysis ranged from 0.60 to 0.67 (mean AUC = 0.63).

Moreover, we further investigated the capability to distinguish PTB from TB in different pregenancy weeks. We also used the 20 characteristic genera screened as the final variables for model prediction and calculated the AUC for different gestational periods. We found that the model in different gestational stages also showed good values, with AUC of 0.726, 0.889, 0.903 for each trimesters, respectively (Fig. 4C).

In order to validate the applicability of the model under different sequencing methods, we first introduce a published external WGS data. WGS data input is based on an RF model constructed at the genus level (20 genera as biomarkers) with an output AUC of 0.738 (Fig. 4D). In addition, we entered the sequencing data collected from 33 subjects into the RF model with an output AUC of 0.638 (Fig. 4E).

### PTB women have unique VM co-abundance network

**Fig. 5:**
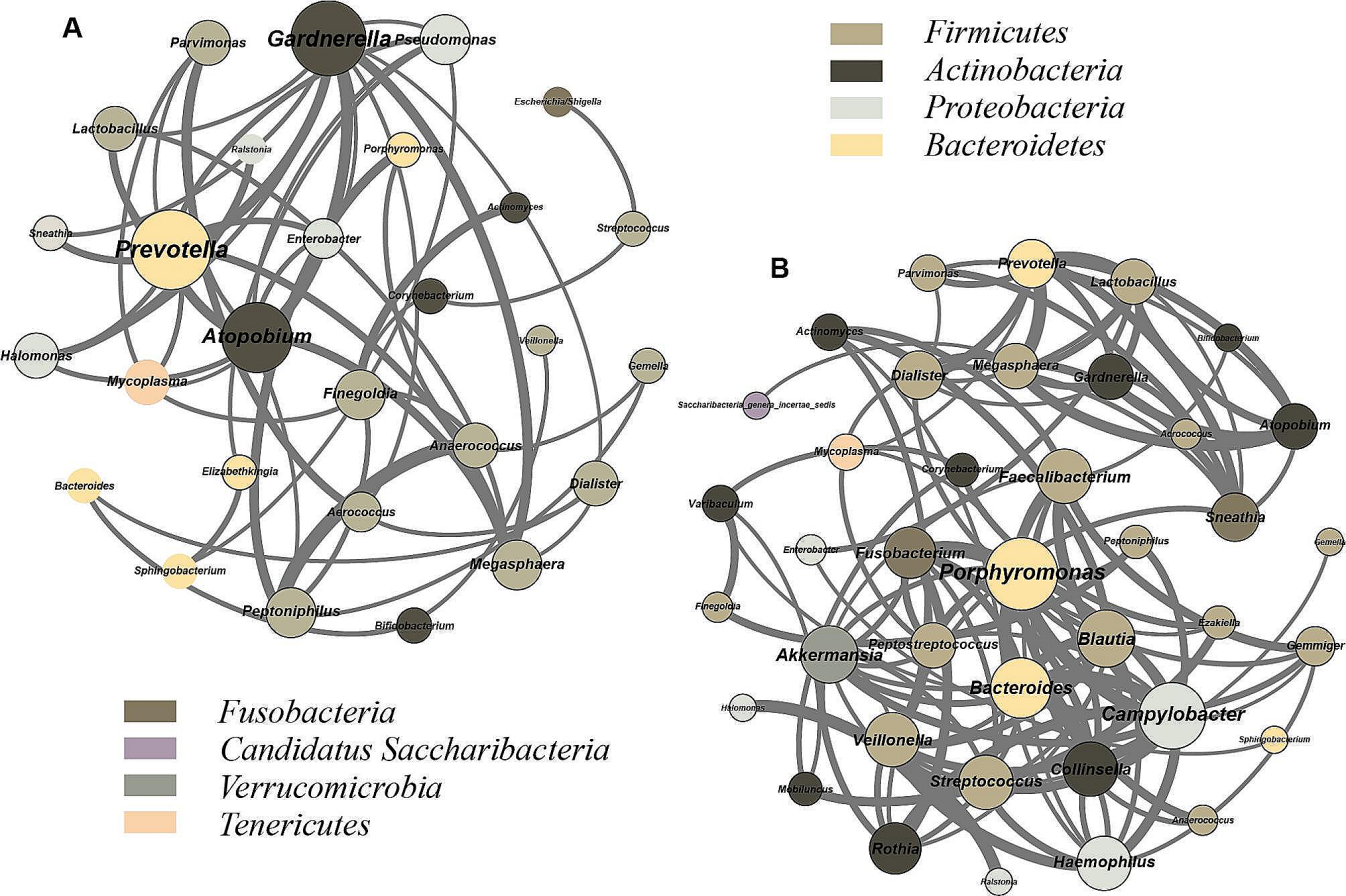
Analysis of vaginal microbiota co-abundance network between preterm and term women. The color of nodes indicates different phylum, node size represents node degree, connecting line indicates the interaction between genera, and width of connecting line represents correlation. (**A**) Microbial co-abundance network in preterm group. (**B**) Microbial co-abundance network in the full-term group

We constructed a network of significantly co-occurring (*r* > 0.7, *P* < 0.05) bacterial families in both groups using Spearman correlation test. We observed that the majority of bacteria in the network belonged to *Firmicutes*, *Actinobacteria* and *Proteobacteria* (Fig. 5A). It can be seen that the network complexity was higher in both TB samples than PTB samples (Fig. 5B). In addition, in the PTB group, we found *Prevotella*, *Gardnerella*, and *Atopobium* as the main hubs and with stronger interactions than in TB.

### Analysis of functional VM pathways in PTB women

**Fig. 6:**
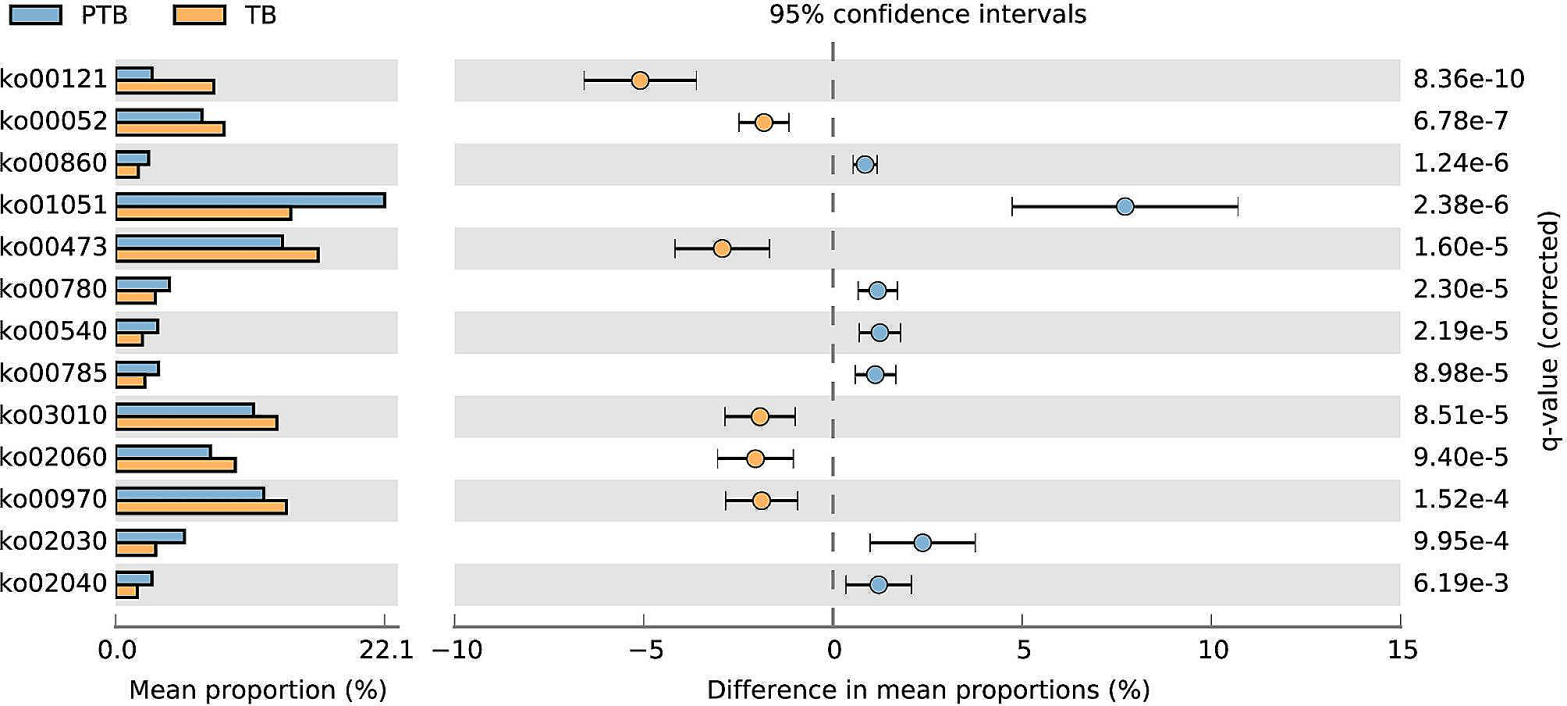
The analysis of functional annotation of the KEGG database combined with the relative abundance of vaginal microbes. Welch’s test shows that preterm brith group is significantly changed in level 3 KEGG pathway

We predicted 165 unique level 3 KEGG Oryhology (KO) pathways in the PTB group versus the TB group vaginal microbiome, of which 79 showed significant intergroup differences. Among them, ko00860, ko01051, ko00780 etc. were enriched in PTB group while ko00121, ko00052, ko00473, etc. were enriched with TB (Fig. 6).

## Discussion

PTB is an important cause of neonatal death, and a non-invasive and accurate early prediction method is urgently needed to reduce the incidence. Based on this, we integrated the VM 16S rRNA-seq data from four different regions of women, explored VM differences, and constructed a robust PTB risk prediction model.

The results of our analysis showed that PTB women have a unique VM profile. In order to exclude the influence of CST on the results, we analyzed data at the CST level. The results showed no significant correlation between CST and PTB rates, indicating that the different CST of women in the four studies had no significant effect on the results. However, this is contrary to the results of some of the current studies [37, 38]. Anne and colleagues found that vaginal CST III or IV was associated with an increased risk of PTB in a African American women study [25]. In the same year, a similar conjecture was made and a positive association between CST IV and PTB was successfully confirmed by researchers [38]. We speculate that the reason for the difference may be due to differences in sample source, size, or a combination of multiple factors. A recent study by Johanna and colleagues may explain some of the differences. The metagenomic community state types they developed make up for the shortcomings of current classification methods in capturing functional information, and their definition of metagenomic subspecies can analyze the composition of vaginal microbiome in a higher dimension, which is missing in our results [39]. In general, the dominance of *Firmicutes* and *Lactobacillus* decreased in PTB, while *Proteobacteria*, *Actinobacteria*, *Bacteroidetes*, etc. were significantly enriched.

Alterations in the abundance of signature microbes potentially trigger PTB. The decrease in the abundance of *Firmicutes*, *Lactobacillus*, is an important sign of dysbiosis in the vaginal environment and is present in almost all gynecological classes of diseases [40–42]. *Lactobacillus* has an irreplaceable role in the vagina, it ensures the acidic environment of the vagina. Intrauterine infections and inflammatory diseases are risk factors for PTB [5], and the decrease in the dominance of *Lactobacilli* in the vagina during pregnancy may accelerate the invasion and upward movement of pathogens [43, 44]. In addition, the role of *Lactobacilli* in immunity cannot be ignored, as immune interference by high-risk bacteria may affect the normal immune function of women during pregnancy, with adverse consequences [45]. Furthermore, the correlation between *Lactobacilli* and gynecological diseases such as BV and HPV infections requires extra caution in the treatment of pregnant women [46], and the impact of the fetus should be taken into account. In general, abundant vaginal *Lactobacilli* in healthy pregnant women may protect the cervicovaginal epithelial barrier, inhibit pathogenic invasion, and modulate the immune response, reducing the incidence of PTB, whereas dysbiosis has the opposite effect. Increased abundance of *Bacteroidetes* was also strongly associated with PTB. Yang-Ah and colleagues detected communities characterized mainly by *Bacteroidetes* and *L. crispatus* in a Korean study only in women with PTB [47], and enrichment of *Bacteroidetes* was present in patients with premature rupture of membranes. In addition, enrichment of gut *Bacteroidetes* was also positively correlated with PTB and detected in the gut of preterm infants [48], amniotic fluid microbes could be its potential source. Interestingly, *Enterobacter* is not a common vaginal bacterium, but is highly represented in both PTB and TB groups, especially in the “Indian” cohort. *Enterobacter* is widespread in nature and its members include commensal gut bacteria (*Enterobacter cloacae*), conditionally pathogenic bacteria and pathogenic bacteria [49]. The presence and increased of *Enterobacter* in the vagina generally represents a dysbiosis and is closely related to clinical infections [50]. We speculate that long-term regular checkups in pregnant women may have contributed to the colonization of *Enterobacter*. The poor health care environment in India compared to other regions may have contributed to the high percentage of *Enterobacter*. In addition, a study found that an increase in *Enterobacter* in women with premature rupture of membranes was positively correlated with downregulation of glycolytic metabolites [51], which has the potential value. Moreover, a study found that *Enterobacter* abundance was negatively correlated with gestational age in fetal fecal and may be involved in the inflammatory response that triggers PTB [52]. The potential origin of *Enterobacter* in feces is amniotic fluid swallowing, and the relationship with maternal VM is self-evident. In conclusion, we suggest that the unconventional composition of VM is a potential risk factor for PTB, and that the immune response to infection caused by pathogenic microbes traveling up the vagina to the amniotic membrane and amniotic fluid may be the most important pathway.

The AUC of the RF model we constructed was 0.877. The biomarkers were extremely similar to the results of Sunwha Park et al. [23], but our AUC was greater. Not only that, the multiperspective model validation, is our relative advantage. The use of microbes can circumvent the disadvantages of current screening methods, and its non-invasive and low cost are its greatest advantages. In addition, future prospective studies using microbial to predict may even advance PTB screening to the preparation stage, which may have an unexpected effect on reducing the incidence worldwide.

We found a unique microbial co-abundance network in PTB women. In addition, the key hubs in the PTB network also present in the PTB prediction model. We can find that the key hubs (*Gardnerella*, *Prevotella*, *Atopobium*, etc.) are closely related to BV [53], and the association between BV and adverse pregnancy outcomes such as PTB [46], miscarriage [54], and premature rupture of membranes [55] has been reported in articles as early as around the 1980s [56]. An experiment in a pregnant mouse model by Luz-Jeannette and colleagues found that vaginal colonization by *G. vaginalis* may induce cervical remodeling and thus PTB by causing local inflammation and inducing an immune response in pregnant mice [57], which may be one of the mechanisms by which BV-associated bacteria induce PTB. A cellular assay showed that *Sneathia* induced upregulation of the secretion of pro-inflammatory cytokines IL-1α, IL-1β and IL-8 in human vaginal epithelial cells, altering the immune metabolic profile and causing local inflammation and tissue damage [58], further demonstrating the harmfulness of BV-associated bacteria. In addition, *Gardnerella* is also associated with a short cervix [13, 59], possibly related to the regulation of human milk oligosaccharides, and the enrichment of *Prevotella*, *Atopobium* in PTB has also been demonstrated [60, 61].

Based on 16S rRNA-seq, PICRUSt was used to infer bacterial community function. The results reveal that changes in VM may lead to significant changes in gene function expression and that these changes may be factors that induce PTB development. We found bacterial chemotaxis was enriched in PTB. Bacterial chemotaxis is defined as the direct movement of bacteria to environmental conditions and is widely distributed among various pathogenic bacteria that cause host infections [62], may be one of the reasons and mechanisms of the rising movement of pathogenic bacteria.

The advantage of this study is that the VM data of PTB women from different regions were pooled for analysis, the sample size was large. Most of the existing PTB prediction models are limited to the model construction, we validated the model by sampled WGS and an external WGS cohorts to ensure the validity and robustness.

This study is not without limits. 16S rRNA-seq analysis methods cannot be accurately annotated to the species, which may have some bias in the bacterial change and model construction. Some studies have shown that there are “PTB-inducing bacteria” in the members of *Lactobacillus* [24]. The elevated *L. iners* may be associated with a short cervix and was found to coexist at a high rate with *G. vaginalis*. In addition, the potential to discover VM presented in vaginal tract is associated with PTB is limited by the relationship between PTB and neonatal infection. Although the newborns were mainly affected and may suffer from neonatal infection, the model was only identified to predict PTB, not for neonatal infection. PTB is a syndrome involving multiple pathological processes, a thorough investigation of the predisposing conditions requires a cross-section of disciplines, the collection of larger and more representative samples, and a focus on individualized differences. Nevertheless, our study provides favorable evidence that VM influence PTB, consolidates the current consensus, extends the membership of ‘risk microbes’, and serves as a useful recommendation for the future noninvasive prediction. Future research should focus on mechanism and investigate how external microorganisms travel up the reproductive tract to the uterus; conduct VM environmental monitoring at an earlier stage and artificially intervene in the dysbiosis vaginal flora of women with pregnancy preparation; and focus on individualized monitoring interventions for more effective clinical application.

In conclusion, the results of this meta-analysis reveal a potential induction of PTB by VM dysbiosis, as evidenced by a decrease in the dominance of *lactobacilli* and an increase in the colonization and prevalence of pathogenic bacteria, but CST in women had little effect. The potential mechanisms may be related to the pathogenic microbes or non-conventional VM composition causing local inflammation, resulting in damage to the protective vaginal barrier, and the upstream movement of microbes to the uterus. Finally, we constructed a PTB prediction model based on 20 differentially characterized genera, which has a high diagnostic value.

## Data Availability

The datasets presented in this study can be found in online repositories. The names of the repository/repositories and accession number(s) can be found below: http://www.ncbi.nlm.nih.gov/bioproject/PRJNA888240.

## References

[1] Liu L, Oza S, Hogan D, Chu Y, Perin J, Zhu J (2016). Global, regional, and national causes of under-5 mortality in 2000–15: an updated systematic analysis with implications for the Sustainable Development Goals. Lancet.

[2] Andrews WW, Goldenberg RL, Mercer B, Iams J, Meis P, Moawad A (2000). The Preterm Prediction Study: association of second-trimester genitourinary chlamydia infection with subsequent spontaneous preterm birth. Am J Obstet Gynecol.

[3] Romero R, Espinoza J, Goncalves LF, Kusanovic JP, Friel L, Hassan S (2007). The role of inflammation and infection in preterm birth. Semin Reprod Med.

[4] Klebanoff MA, Brotman RM (2018). Treatment of bacterial vaginosis to prevent preterm birth. Lancet.

[5] Goldenberg RLCJ, Iams JD, Romero R (2008). Epidemiology and causes of preterm birth. Lancet.

[6] Marret SAP, Marpeau L, Marchand L, Pierrat V, Larroque B, Foix-L’Hélias L, Thiriez G, Fresson J, Alberge C, Rozé JC, Matis J, Bréart G, Kaminski M (2007). Epipage Study Group. Neonatal and 5-year outcomes after birth at 30–34 weeks of gestation. Obstet Gynecol.

[7] Rofael SAD, McHugh TD, Troughton R, Beckmann J, Spratt D, Marlow N et al. Airway microbiome in adult survivors of extremely preterm birth: the EPICure study. Eur Respir J. 2019;53(1). 10.1183/13993003.01225-2018 30464016

[8] Lobel M, Cannella DL, Graham JE, DeVincent C, Schneider J, Meyer BA (2008). Pregnancy-specific stress, prenatal health behaviors, and birth outcomes. Health Psychol.

[9] Chee WJY, Chew SY, Than LTL (2020). Vaginal microbiota and the potential of Lactobacillus derivatives in maintaining vaginal health. Microb Cell Fact.

[10] Witkin SS, Linhares IM (2017). Why do lactobacilli dominate the human vaginal microbiota?. BJOG.

[11] Donati L, Di Vico A, Nucci M, Quagliozzi L, Spagnuolo T, Labianca A (2010). Vaginal microbial flora and outcome of pregnancy. Arch Gynecol Obstet.

[12] Brotman RM, Ravel J, Bavoil PM, Gravitt PE, Ghanem KG (2014). Microbiome, sex hormones, and immune responses in the reproductive tract: challenges for vaccine development against sexually transmitted infections. Vaccine.

[13] Gerson KD, McCarthy C, Elovitz MA, Ravel J, Sammel MD, Burris HH (2020). Cervicovaginal microbial communities deficient in Lactobacillus species are associated with second trimester short cervix. Am J Obstet Gynecol.

[14] Iams JDGR, Meis PJ, Mercer BM, Moawad A, Das A, Thom E, McNellis D, Copper RL, Johnson F, Roberts JM (1996). The length of the cervix and the risk of spontaneous premature delivery. National Institute of Child Health and Human Development Maternal Fetal Medicine Unit Network. N Engl J Med.

[15] Payne MS, Bayatibojakhi S. Exploring Preterm Birth as a Polymicrobial Disease: an overview of the uterine microbiome. Front Immunol. 2014;5. 10.3389/fimmu.2014.00595 PMC4245917 25505898

[16] DiGiulio DB (2012). Diversity of microbes in amniotic fluid. Semin Fetal Neonatal Med.

[17] Gardella CRD, Hitti J, Agnew K, Krieger JN, Eschenbach D (2004). Identification and sequencing of bacterial rDNAs in culture-negative amniotic fluid from women in premature labor. Am J Perinatol.

[18] Krohn MAHS, Nugent RP, Cotch MF, Carey JC, Gibbs RS, Eschenbach DA (1995). The genital flora of women with intraamniotic infection. Vaginal infection and Prematurity Study Group. J Infect Dis.

[19] Leitich H, Bodner-Adler B, Brunbauer M, Kaider A, Egarter C, Husslein P (2003). Bacterial vaginosis as a risk factor for preterm delivery: a meta-analysis. Am J Obstet Gynecol.

[20] Hillier SLNR, Eschenbach DA, Krohn MA, Gibbs RS, Martin DH, Cotch MF, Edelman R, Pastorek JG, Rao AV (1995). Association between bacterial vaginosis and preterm delivery of a low-birth-weight infant. The vaginal infections and Prematurity Study Group. N Engl J Med.

[21] Romero RHS, Gajer P, Tarca AL, Fadrosh DW, Nikita L, Galuppi M, Lamont RF, Chaemsaithong P, Miranda J, Chaiworapongsa T, Ravel J (2014). The composition and stability of the vaginal microbiota of normal pregnant women is different from that of non-pregnant women. Microbiome.

[22] de Freitas AS, Dobbler PCT, Mai V, Procianoy RS, Silveira RC, Corso AL (2020). Defining microbial biomarkers for risk of preterm labor. Braz J Microbiol.

[23] Park S, Moon J, Kang N, Kim YH, You YA, Kwon E (2022). Predicting preterm birth through vaginal microbiota, cervical length, and WBC using a machine learning model. Front Microbiol.

[24] Kumar S, Kumari N, Talukdar D, Kothidar A, Sarkar M, Mehta O (2021). The Vaginal Microbial signatures of Preterm Birth Delivery in Indian Women. Front Cell Infect Microbiol.

[25] Dunlop AL, Satten GA, Hu YJ, Knight AK, Hill CC, Wright ML (2021). Vaginal Microbiome composition in early pregnancy and risk of spontaneous Preterm and Early Term Birth among African American Women. Front Cell Infect Microbiol.

[26] Fudaba M, Kamiya T, Tachibana D, Koyama M, Ohtani N. Bioinformatics analysis of oral, vaginal, and rectal microbial profiles during pregnancy: a pilot study on the bacterial co-residence in pregnant women. Microorganisms. 2021;9(5). 10.3390/microorganisms9051027 PMC8151423 34064634

[27] Odogwu NM, Chen J, Onebunne CA, Jeraldo P, Yang L, Johnson S et al. Predominance of Atopobium vaginae at Midtrimester: a potential Indicator of Preterm Birth Risk in a Nigerian cohort. mSphere. 2021;6(1). 10.1128/mSphere.01261-20 PMC7885325 33504666

[28] Feehily C, Crosby D, Walsh CJ, Lawton EM, Higgins S, McAuliffe FM (2020). Shotgun sequencing of the vaginal microbiome reveals both a species and functional potential signature of preterm birth. NPJ Biofilms Microbiomes.

[29] Rognes T, Flouri T, Nichols B, Quince C, Mahe F (2016). VSEARCH: a versatile open source tool for metagenomics. PeerJ.

[30] Liu X, Cao Y, Xie X, Qin X, He X, Shi C (2021). Association between vaginal microbiota and risk of early pregnancy miscarriage. Comp Immunol Microbiol Infect Dis.

[31] Ravel J, Gajer P, Abdo Z, Schneider GM, Koenig SS, McCulle SL (2011). Vaginal microbiome of reproductive-age women. Proc Natl Acad Sci U S A.

[32] Gajer P, Brotman RM, Bai G, Sakamoto J, Schutte UM, Zhong X (2012). Temporal dynamics of the human vaginal microbiota. Sci Transl Med.

[33] Edgar RC (2010). Search and clustering orders of magnitude faster than BLAST. Bioinformatics.

[34] Wu Y, Jiao N, Zhu R, Zhang Y, Wu D, Wang AJ (2021). Identification of microbial markers across populations in early detection of colorectal cancer. Nat Commun.

[35] Segata N, Izard J, Waldron L, Gevers D, Miropolsky L, Garrett WS (2011). Metagenomic biomarker discovery and explanation. Genome Biol.

[36] Assenov Y, Ramirez F, Schelhorn SE, Lengauer T, Albrecht M (2008). Computing topological parameters of biological networks. Bioinformatics.

[37] Callahan BJ, DiGiulio DB, Goltsman DSA, Sun CL, Costello EK, Jeganathan P (2017). Replication and refinement of a vaginal microbial signature of preterm birth in two racially distinct cohorts of US women. Proc Natl Acad Sci U S A.

[38] Florova V, Romero R, Tarca AL, Galaz J, Motomura K, Ahmad MM (2021). Vaginal host immune-microbiome interactions in a cohort of primarily African-American women who ultimately underwent spontaneous preterm birth or delivered at term. Cytokine.

[39] Holm JB, France MT, Gajer P, Ma B, Brotman RM, Shardell M, Forney L, Ravel J (2023). Integrating compositional and functional content to describe vaginal microbiomes in health and disease. Microbiome.

[40] Laniewski P, Barnes D, Goulder A, Cui H, Roe DJ, Chase DM (2018). Linking cervicovaginal immune signatures, HPV and microbiota composition in cervical carcinogenesis in non-hispanic and hispanic women. Sci Rep.

[41] Wahid M, Dar SA, Jawed A, Mandal RK, Akhter N, Khan S et al. Microbes in gynecologic cancers: causes or consequences and therapeutic potential. Semin Cancer Biol. 2021. 10.1016/j.semcancer.2021.07.013 34302959

[42] Anahtar MN, Gootenberg DB, Mitchell CM, Kwon DS (2018). Cervicovaginal Microbiota and Reproductive Health: the Virtue of simplicity. Cell Host Microbe.

[43] Younes JA, Lievens E, Hummelen R, van der Westen R, Reid G, Petrova MI (2018). Women and their microbes: the unexpected friendship. Trends Microbiol.

[44] Anton L, Sierra LJ, DeVine A, Barila G, Heiser L, Brown AG (2018). Common Cervicovaginal Microbial supernatants alter cervical epithelial function: mechanisms by which Lactobacillus crispatus contributes to Cervical Health. Front Microbiol.

[45] Nicolò S, Tanturli M, Mattiuz G, Antonelli A, Baccani I, Bonaiuto C et al. Vaginal Lactobacilli and Vaginal Dysbiosis-Associated Bacteria differently affect cervical epithelial and Immune Homeostasis and Anti-viral defenses. Int J Mol Sci. 2021;22(12). 10.3390/ijms22126487 PMC8234132 34204294

[46] Lamont RF (2015). Advances in the Prevention of infection-related Preterm Birth. Front Immunol.

[47] You YA, Kwon EJ, Choi SJ, Hwang HS, Choi SK, Lee SM (2019). Vaginal microbiome profiles of pregnant women in Korea using a 16S metagenomics approach. Am J Reprod Immunol.

[48] Yin C, Chen J, Wu X, Liu Y, He Q, Cao Y (2021). Preterm Birth is correlated with increased oral originated Microbiome in the gut. Front Cell Infect Microbiol.

[49] Davin-Regli A, Lavigne JP, Pages JM. Enterobacter spp.: update on taxonomy, clinical aspects, and emerging Antimicrobial Resistance. Clin Microbiol Rev. 2019;32(4). 10.1128/CMR.00002-19 PMC6750132 31315895

[50] Shao Y, Forster SC, Tsaliki E, Vervier K, Strang A, Simpson N (2019). Stunted microbiota and opportunistic pathogen colonization in caesarean-section birth. Nature.

[51] Liu L, Chen Y, Chen JL, Xu HJ, Zhan HY, Chen Z (2021). Integrated metagenomics and metabolomics analysis of third-trimester pregnant women with premature membrane rupture: a pilot study. Ann Transl Med.

[52] Ardissone AN, de la Cruz DM, Davis-Richardson AG, Rechcigl KT, Li N, Drew JC (2014). Meconium microbiome analysis identifies bacteria correlated with premature birth. PLoS ONE.

[53] Chen X, Lu Y, Chen T, Li R (2021). The female vaginal microbiome in Health and bacterial vaginosis. Front Cell Infect Microbiol.

[54] CA S (1991). Bacterial vaginosis. Clin Microbiol Rev.

[55] Yan C, Hong F, Xin G, Duan S, Deng X, Xu Y (2022). Alterations in the vaginal microbiota of patients with preterm premature rupture of membranes. Front Cell Infect Microbiol.

[56] Hay PELR, Taylor-Robinson D, Morgan DJ, Ison C, Pearson J (1994). Abnormal bacterial colonisation of the genital tract and subsequent preterm delivery and late miscarriage. BMJ.

[57] Sierra LJ, Brown AG, Barila GO, Anton L, Barnum CE, Shetye SS (2018). Colonization of the cervicovaginal space with Gardnerella vaginalis leads to local inflammation and cervical remodeling in pregnant mice. PLoS ONE.

[58] McKenzie R, Maarsingh JD, Laniewski P, Herbst-Kralovetz MM (2021). Immunometabolic Analysis of Mobiluncus mulieris and Eggerthella sp. Reveals Novel insights into their pathogenic contributions to the hallmarks of bacterial vaginosis. Front Cell Infect Microbiol.

[59] Di Paola M, Seravalli V, Paccosi S, Linari C, Parenti A, De Filippo C et al. Identification of Vaginal Microbial communities Associated with Extreme cervical shortening in pregnant women. J Clin Med. 2020;9(11). 10.3390/jcm9113621 PMC7698214 33182750

[60] Pausan MR, Kolovetsiou-Kreiner V, Richter GL, Madl T, Giselbrecht E, Obermayer-Pietsch B et al. Human milk oligosaccharides modulate the risk for Preterm Birth in a Microbiome-Dependent and -independent manner. mSystems. 2020;5(3). 10.1128/mSystems.00334-20 PMC7289590 32518196

[61] Fettweis JM, Serrano MG, Brooks JP, Edwards DJ, Girerd PH, Parikh HI (2019). The vaginal microbiome and preterm birth. Nat Med.

[62] Keegstra JM, Carrara F, Stocker R (2022). The ecological roles of bacterial chemotaxis. Nat Rev Microbiol.

